# Pandemic-driven immune imprinting accelerates evolution of human coronavirus OC43

**DOI:** 10.1371/journal.pntd.0014109

**Published:** 2026-03-17

**Authors:** Shuiping Lu, Qi Shen, Huanru Wang, Mingyi Cai, Jingjing Hu, Yi Li, Yuxi Wang, Feng Yuan, Qingyuan Xu, Weijie Chen, Yitian Wu, Jiasheng Xiong, Zheng Teng, Mengting Tang, Chenglong Xiong

**Affiliations:** 1 School of Public Health, Fudan University, Key Lab of Public Health Safety, Ministry of Education, Shanghai, China; 2 Shanghai Jing’an District Center for Disease Control and Prevention (Shanghai Jing’an District Health Supervision Institute), Shanghai, China; 3 Pathogen Testing Center, Shanghai Municipal Center for Disease Control and Prevention (Shanghai Academy of Preventive Medicine), Shanghai, China; 4 Shanghai Institute of Infectious Disease and Biosecurity, Fudan University, Shanghai, China; 5 Shanghai Municipal Center for Disease Control and Prevention, Shanghai, China; Central University of Tamil Nadu, INDIA

## Abstract

Global pandemic interventions have reshaped host-virus dynamics, potentially altering the evolution of endemic pathogens. Here, we report accelerated genomic evolution of human coronavirus OC43 (HCoV-OC43)—a close relative of pandemic-associated coronaviruses—following recent worldwide epidemiological shifts. Bayesian analysis of longitudinal surveillance data revealed a 3.76-fold increase (8.9403 × 10 ⁻ ⁴ nucleotide substitutions/site/year, 95% HPD: 4.9075 × 10 ⁻ ⁴, 1.3053 × 10 ⁻ ³) in the spike gene substitution rate of the currently dominant genotype K post-2020. Positively selected mutations were mainly located in the spike protein, and some colocalize with antigenic epitopes. Crucially, structural modeling demonstrated that broadly neutralizing antibodies targeting conserved stem-helix (S2P6) and fusion-peptide (COV44–62/79, 76E1) epitopes of high-pathogenicity betacoronaviruses cross-bind HCoV-OC43 spike protein, establishing a mechanistic basis for immune-driven selection. These findings suggest that population-level immune imprinting may play a potential driving role in mutations within key domains of HCoV-OC43, although further validation is required. Sustained co-surveillance of co-circulating coronaviruses is imperative to anticipate emergent variants with altered pathogenicity.

## Author summary

Since the global outbreak of COVID-19, extensive public health interventions—such as mask wearing, social distancing, and travel restrictions—along with widespread vaccination and antiviral treatments, have profoundly influenced the transmission dynamics of various respiratory viruses. HCoV-OC43 and SARS-CoV-2 both belong to the genus *Betacoronavirus* and share structural and antigenic similarities, which may lead to potential cross-reactivity and cross-protective immunity between them. The pandemic may have exerted immune selection pressures that drive adaptive mutations in HCoV-OC43, enabling it to maintain endemic circulation. However, current research on the molecular basis of cross-immunity between these coronaviruses and its impact on viral evolution remains limited. By comparing substitution rates and analyzing antigen–antibody interactions, our study revealed an accelerated evolutionary trend of HCoV-OC43 under potential immune selection pressure. These findings underscore the importance of ongoing joint surveillance of co-circulating coronaviruses to anticipate the emergence of new variants with altered pathogenicity.

## Introduction

In 1967, McIntosh and his colleagues first isolated human coronavirus OC43 (HCoV-OC43) from organ cultures of human embryonic trachea obtained from patients with respiratory disease [1]. Among the seven coronaviruses known to infect humans, SARS-CoV, MERS-CoV, and SARS-CoV-2 are highly pathogenic, capable of causing large-scale outbreaks and fatal pneumonia. In contrast, HCoV-OC43, HCoV-229E, HCoV-NL63, and HCoV-HKU1 are endemic seasonal coronaviruses that typically cause mild upper respiratory tract illness [2,3]. Compared to the other three common endemic seasonal coronaviruses, HCoV-OC43 exhibits a relatively higher prevalence of respiratory tract infections in children and adults. Furthermore, novel genotypes of HCoV-OC43 are continuously being identified, posing a considerable threat to immunocompromised individuals, infants, and the elderly [4–12]. The genome of HCoV-OC43, similar to that of SARS-CoV-2, is a positive-sense single-stranded RNA (+ssRNA) approximately 30.7 kb in length, with the first two-thirds region encoding the ORF1ab polyprotein (ORF1ab) and the remaining one-third encoding structural proteins and non-structural proteins, including the spike surface glycoprotein (S), envelope protein (E), membrane protein (M), nucleocapsid protein (N), hemagglutinin-esterase (HE), non-structural protein 2 (ns2), and non-structural protein 12.9 (ns12.9) [13–16].

The emergence of SARS-CoV-2 was first observed at the end of December 2019, subsequently giving rise to a global pandemic with far-reaching implications for human health and the advancement of medical research on coronaviruses. Given its low risk and structural similarity, HCoV-OC43 is often used as a low-risk model to study SARS-CoV-2 [17]. By seroepidemiology, Thoisy et al. found that the long-term endemic equilibrium of seasonal coronaviruses is the result of a dynamic balance among increasing population immunity through new infections, waning antibody levels, and the introduction of newly susceptible children [18]. Furthermore, several studies have found cross-reactivity and partial cross-protective immunity between SARS-CoV-2 and endemic coronaviruses such as HCoV-OC43 in host immune cells, owing to frequent reinfections and widespread vaccination [19,20].

SARS-CoV-2 has been circulating for over five years, in contrast to HCoV-OC43, which has persisted for more than fifty years. The close phylogenetic relationship between the two viruses, along with their localized similarity in the primary or higher-order structures of several proteins, strongly suggests the potential for antibody cross-reactivity. Consequently, the SARS-CoV-2 pandemic may have disrupted the long-term endemic equilibrium of HCoV-OC43 and accelerated its evolution, particularly under immune selection pressure. In this context, we performed genotyping of HCoV-OC43 based on phylogenetic analysis using whole genome (WG) sequences as well as full-length S, RNA-dependent RNA polymerase (RdRp), and N gene sequences. Across these four genetic levels, we then estimated and compared the nucleotide substitution rates of the predominant genotypes before and after the pandemic and conducted selection pressure analyses. Moreover, at the genetic level of the mutation-prone spike protein, we analyzed the historical evolutionary patterns of positively selected sites, assessed polymorphism of amino acid variation sites, predicted both linear and conformational B-cell epitopes, and evaluated antigen–antibody binding interactions. These analyses aimed to explore the mechanisms of the sustained epidemic of HCoV-OC43 and the potential for cross-reactivity between SARS-CoV-2 and HCoV-OC43. This study provides insights for the prevention of future outbreaks of HCoV-OC43 and other related coronaviruses.

## Results

### Results of HCoV-OC43 genotyping

**Fig 1:**
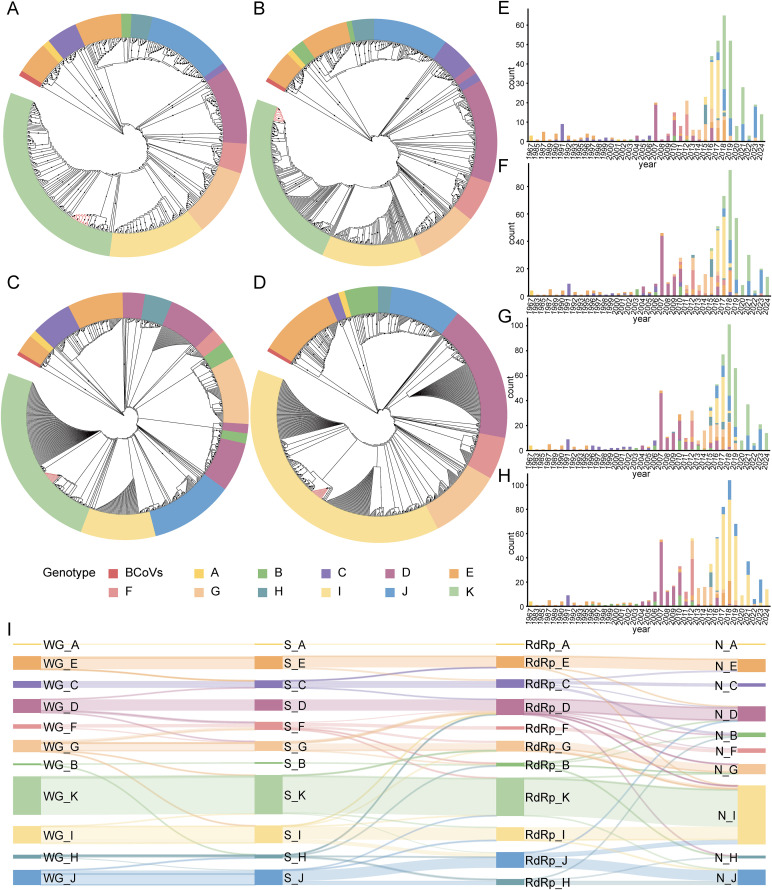
Summary results of HCoV-OC43 viral genotyping. **A-D**. genotyping results based on the WG, S, RdRp, and N gene sequences of HCoV-OC43 (**A**. WG genotyping results; **B**. S genotyping results; **C**. RdRp genotyping results; **D**. N genotyping results). The black dots on the branches indicate bootstrap values ≥ 70. Branches marked in red on the evolutionary tree indicate sequences obtained by sequencing in this study. **E-H**. distribution results of sequence collection years for different genotypes based on WG, S, RdRp, and N genotyping of HCoV-OC43 (**E**. distribution results based on WG genotyping; **F**. distribution results based on S genotyping; **G**. distribution results based on RdRp genotyping; **H**. distribution results based on N genotyping). **I**. WG, S, RdRp, and N genotyping results of the same virus strain of HCoV-OC43.

HCoV-OC43 exhibited distinct predominant genotypes during different periods (Fig 1A-1H). At the WG level, the predominant genotypes shifted over time prior to the emergence of SARS-CoV-2, from genotype A (1967) to genotype E (1985–1990), genotype C (1991–2006), genotype D (2007–2010), genotype F (2011–2013), genotype G (2014–2015), genotype H (2015), genotype I (2016–2017), and genotype K (2018–2019). Following the emergence and subsequent pandemic of SARS-CoV-2, genotypes J and K (behaving as genotype I at the N-gene level) have become the predominant genotypes, with genotype K accounting for the majority of recent viral isolates. Fourteen sequences obtained by sequencing (PV798427-PV798440) in this study were all classified as genotype K. Notably, all HCoV-OC43 strains collected since 2024 belong to genotype K, indicating that it is highly likely to occupy the currently dominant position (Fig 1E). Besides, comparison of phylogenetic trees constructed from different gene regions for the same viral strains revealed that, except for genotype A, strains assigned to other genotypes appeared in partially inconsistent phylogenetic positions across the maximum likelihood (ML) trees based on WG, S, RdRp, and N genes (Fig 1I). Detailed summary results of the same virus strains under the four genotyping levels (WG, S, RdRp, and N) can be found in S1 Table.

### Bayesian evolutionary analysis

**Fig 2:**
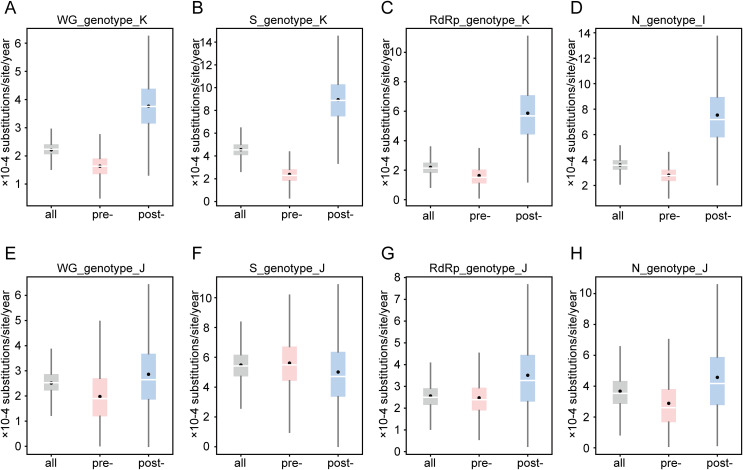
Nucleotide substitution rates of HCoV-OC43 genotypes K and J at different gene levels. **A-D**. nucleotide substitution rates of genotype K at the WG, S, RdRp, and N gene levels (expressed at the N gene level as genotype I). **E-H**. nucleotide substitution rates of genotype J at the WG, S, RdRp, and N gene levels. The all, pre-pandemic (pre-), and post-pandemic (post-) collected sequences from each dataset were imported into the BEAST software for analysis separately. The white line in the box plot indicates the median, and the black dot indicates the mean.

For genotype K, which currently occupies a dominant position after the emergence of SARS-CoV-2, the overall nucleotide substitution rate at the WG level was estimated at 2.2156 × 10 ⁻ ⁴ nucleotide substitutions/site/year (95% HPD: 1.7252 × 10 ⁻ ⁴, 2.7172 × 10 ⁻ ⁴), and the time to the most recent common ancestor (tMRCA) traced to approximately mid-June 2010 (2010.4476; 95% HPD: 2002.1353, 2015.0588) (S2 Table). Comparison of total nucleotide substitution rates across the S, RdRp, and N genes revealed that the S gene exhibited the highest rate, whereas the RdRp gene showed the slowest (S2 Table and Fig 2A-2D). Furthermore, by comparing the nucleotide substitution rates of the WG, S, RdRp, and N genes before and after the SARS-CoV-2 pandemic, there was an accelerated evolutionary trend in all four genetic levels. The acceleration was most pronounced in the S gene, with nucleotide substitution rates increasing from 2.3757 × 10 ⁻ ⁴ (95% HPD: 9.4851 × 10 ⁻ ⁵, 3.9034 × 10 ⁻ ⁴) to 8.9403 × 10 ⁻ ⁴ (95% HPD: 4.9075 × 10 ⁻ ⁴, 1.3053 × 10 ⁻ ³) (S2 Table and Fig 2A-2D).

For genotype J, which was in subdominant position after the emergence of SARS-CoV-2, the WG-level overall nucleotide substitution rate was 2.5133 × 10 ⁻ ⁴ nucleotide substitutions/site/year (95% HPD: 1.5595 × 10 ⁻ ⁴, 3.4601 × 10 ⁻ ⁴), with the tMRCA estimated at mid-January 2006 (2006.0498; 95% HPD: 1995.1384, 2013.8474) (S2 Table). A similar pattern was observed, with the S gene evolving fastest and RdRp slowest (S2 Table and Fig 2E-2H). However, in contrast to genotype K, post-pandemic analysis revealed accelerated evolution in the WG, RdRp, and N genes, whereas the S gene showed a deceleration in its nucleotide substitution rates (S2 Table and Fig 2E-2H).

Besides, recombination analysis identified only a few potential recombinant sequences, all of which were sampled before the COVID-19 pandemic, and SimPlot validation did not reveal clear or stable parental fragment exchanges (detailed information is in S1 File). Therefore, the impact of recombination on the BEAST-based estimation of nucleotide substitution rates is expected to be minimal.

### Selection pressure analysis and historical evolution analysis of positively selected sites

Positively selected sites were found only in the S and N proteins of genotype K (behaving as genotype I at the N gene level) and in the S protein of genotype J (S3 Table). No positively selected sites were detected in the remaining proteins. Specifically, seventeen positively selected sites (identified by at least one method) were detected in the S protein of genotype K: twelve—sites 25, 26, 38, 40, 67, 89, 195, 199, 265, 266, 270, and 271—were located in the N-terminal domain (NTD) of the S1 subunit; two—sites 504 and 506—were in the C-terminal domain (CTD) of the S1 subunit; one—site 900—was in the fusion peptide (FP) region of the S2 subunit; two—sites 1251 and 1338—were in the other region (site 1251 was mapped to the stem helix region) of the S protein (S3 Table). In the N protein of genotype K, three positively selected sites were identified at sites 49, 79, and 248 (S3 Table). In genotype J, nine positively selected sites (identified by at least one method) were detected. Among these, six positively selected sites—454, 472, 481, 503, 554, and 573—were identified in the CTD of the S1 subunit (S3 Table).

**Fig 3:**
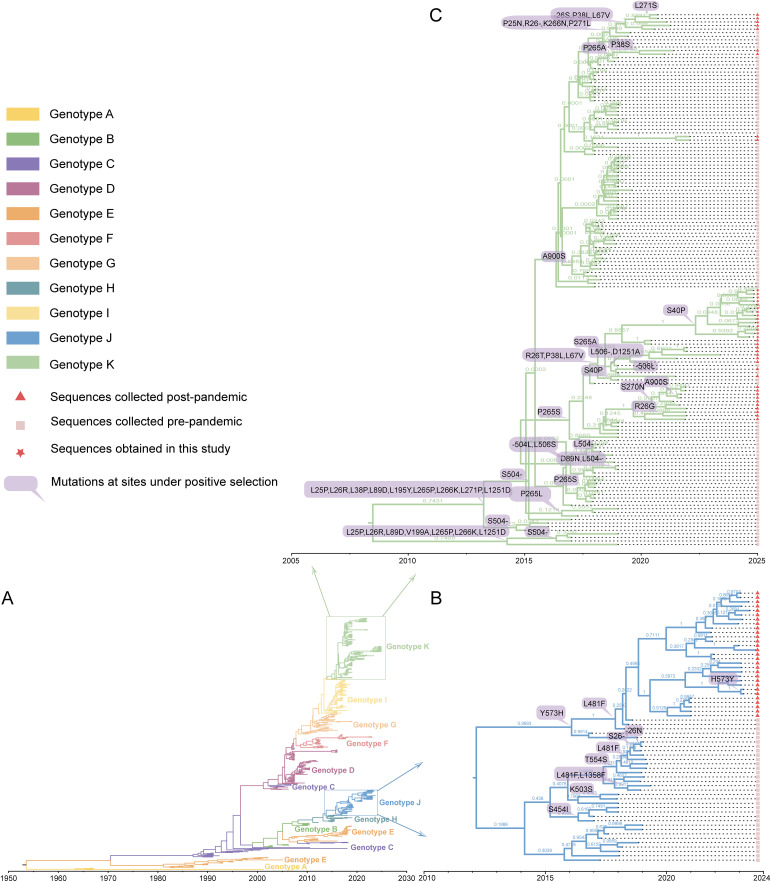
MCC trees labelled with positively selected amino acid mutations of the S protein. **A**. MCC tree of all S protein gene sequences. Different genotypes are marked in different colors. **B-C**. MCC tree of genotypes J and K. Branch support values represent Posterior Probability. The tree nodes are labelled with amino acid mutations of positively selected sites. Red triangles represent sequences collected post-pandemic, pink squares represent sequences collected pre-pandemic, and red pentagrams represent sequences obtained by sequencing in this study.

The results of historical evolution analysis showed that amino acid mutations at positively selected sites almost all occurred along internal nodes of the time-scaled maximum clade credibility (MCC) trees. Lots of mutations at positively selected sites in genotype K, including R26G/T, P38L/S, S40P, L67V, S265A, S270N, L271S, and D1251A, were observed at tree nodes or within subclades corresponding to sequences collected after the SARS-CoV-2 pandemic. In contrast, the positively selected sites in genotype J were primarily associated with tree nodes corresponding to pre-pandemic sequences (Fig 3).

Furthermore, the ML trees with branch lengths in units of substitutions per site reconstructed by IQ-TREE showed that within genotype K, sequences sampled after 2020 accumulated more substitutions compared with those sampled before 2020, whereas genotype J exhibited the reverse pattern (S1 Fig). This provided qualitative, model-light support that was basically consistent with the accelerated evolutionary patterns inferred from our Beast analyses and largely in agreement with our historical evolution analysis of positively selected sites (Figs 2, 3 and S1).

### Polymorphism analysis of amino acid variation sites

Three amino acid variation sites were identified in the S protein of genotype J and six in that of genotype K (Table 1). For genotype J, the major amino acid usage frequency at variation site 481 showed a statistically significant difference before and after the pandemic (P < 0.01); variation sites 481 and 573 coincided with positively selected sites (Table 1). For genotype K, all six variation sites—26, 38, 44, 67, 265, and 483—exhibited significant differences in the major amino acid usage frequencies (P < 0.01), among which sites 26, 38, 67, and 265 also corresponded to positively selected sites (Table 1).

**Table 1:** Results of amino acid variation site analysis of the spike protein of HCoV-OC43.

Genotype	Site^a^	Region^b^	Majority or not	Pre-pandemic	Post-pandemic	P	Mutation
J	33	S1_NTD	+	30 (90.9%) [30N]	29 (100.0%) [29S]	P = 0.284	N33S
			–	3 (9.1%) [3S]	0 (0.0%)		
	481**	S1_CTD	+	22 (66.7%) [22L]	29 (100.0%) [29F]	P = 0.001	L481F
			–	11 (33.3%) [11F]	0 (0.0%)		
	573	S1_CTD	+	27 (81.8%) [27Y]	27 (93.1%) [27H]	P = 0.346	Y573H
			–	6 (18.2%) [6H]	2 (6.9%) [2Y]		
K	26 ***	S1_NTD	+	102 (95.3%) [102R]	23 (54.8%) [23T]	P < 0.001	R26T
			–	5 (4.7%) [3-:2T]	19 (45.2%) [10R:5-:2G:2S]		
	38 ***	S1_NTD	+	99 (92.5%) [99P]	28 (66.7%) [28L]	P < 0.001	P38L
			–	8 (7.5%) [8L]	14 (33.3%) [12P:2S]		
	44 ***	S1_NTD	+	104 (97.2%) [104D]	27 (64.3%) [27E]	P < 0.001	D44E
			–	3 (2.8%) [3E]	15 (35.7%) [15D]		
	67 ***	S1_NTD	+	104 (97.2%) [104L]	28 (66.7%) [28V]	P < 0.001	L67V
			–	3 (2.8%) [3V]	14 (33.3%) [14L]		
	265 **	S1_NTD	+	87 (81.3%) [87P]	24 (57.1%) [24A]	P = 0.002	P265A, P265S
			–	20 (18.7%) [11S:4L:3A:2R]	18 (42.9%) [11S:5-:2P]		
	483***	S1_CTD	+	107 (100.0%) [107N]	26 (61.9%) [26D]	P < 0.001	N483D
			–	0 (0.0%)	16 (38.1%) [16N]		

^a^Statistical significance was assessed using the chi-square test: “**” indicates 0.001 ≤ P < 0.01; “***” indicates P < 0.001. Sites were considered significant at P < 0.01.

^b^The delineation of gene regions was based on the YP_009555241.1 sequence (NCBI database) and the P36334 sequence (UniProt database, https://www.uniprot.org/).

### Prediction of potential linear and conformational B-cell epitopes

In the β-turn and random coil regions, which are typically enriched for antigenic epitopes, seven potential linear B-cell epitopes were predicted in each of the S proteins of HCoV-OC43 genotypes J and K, with two epitopes shared between them, resulting in a total of twelve unique predicted potential linear B-cell epitopes (S2 Fig and S4 Table). Notably, in genotype K, sites 265, 266, 270, and 271 in the NTD of the S1 subunit, identified as both positively selected sites and predicted linear epitope sites, were located within the epitope 264-RPKDGFSP-271. Among these, site 265 was also identified as a statistically significant variation site. Positively selected site 472 was identified in the predicted linear epitope 472-VFKPQP-477 of genotype J (Tables 1, S3 and S4).

**Fig 4:**
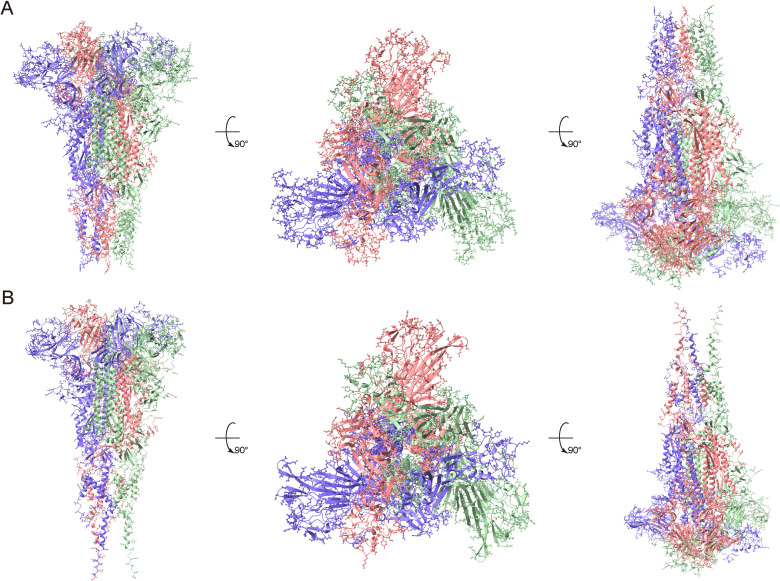
Potential B-cell conformational epitopes on the S protein of HCoV-OC43 genotypes J and K. **A-B**. potential B-cell conformational epitopes of genotypes J and K on the S protein displayed in three orthogonal views (**A**. genotype J; **B**. genotype K). Potential B-cell conformational epitopes were predicted by Discotope-3.0 based on the AlphaFold-predicted S protein structures. Three chains of the HCoV-OC43 spike protein are depicted in purple, pink, and green cartoons, with potential epitope sites represented as ball-and-stick atoms.

Based on AlphaFold-predicted three-dimensional (3D) structures of the S proteins of genotypes J and K, 447 and 418 potential conformational B-cell epitope residues were predicted, respectively (S5 Table). These residues were distributed across multiple regions of the S protein, including the NTD and CTD of the S1 subunit, the S1/S2 cleavage region, and the fusion peptide region of the S2 subunit (Fig 4 and S5 Table). Among them, the positively selected sites (sites 25, 26, 38, 89, 195, 199, 265 and 266) and statistically significant variation sites (sites 26, 38, 44, and 265) in the NTD of the S1 subunit of genotype K were also mapped to predicted conformational epitope residues; two positively selected sites 26 (25) and 44 (43) were identified within the predicted conformational epitope residues of genotype J (Tables 1, S3 and S5). Here, 25 and 43 in parentheses indicate the aligned conformational epitope sites.

### Antigen–antibody binding analysis

The 9-O-acetylated sialic acid (9-O-Ac-Sia) receptor (PDB: 6NZK) binds to the S1 NTD of the HCoV-OC43 S protein, inserting its acetyl group into two hydrophobic binding pockets, P1 and P2, which defined by the LI loop (27–NDKDTG–32) and the L2 loop (80–LKGSVLL–86) and separated by Trp90 (S3A-S3C Fig). Of the positively selected sites identified in the S protein of genotype K, sites 38 (33) and 40 (35) are situated near the binding region, whereas site 89 (84) resides directly within it, suggesting potential functional relevance (S3C Fig). Here, 38 (33) indicates that 38 represents the positively selected site, while 33 corresponds to the aligned position in the sequence used for the 6NZK structural prediction.

**Fig 5:**
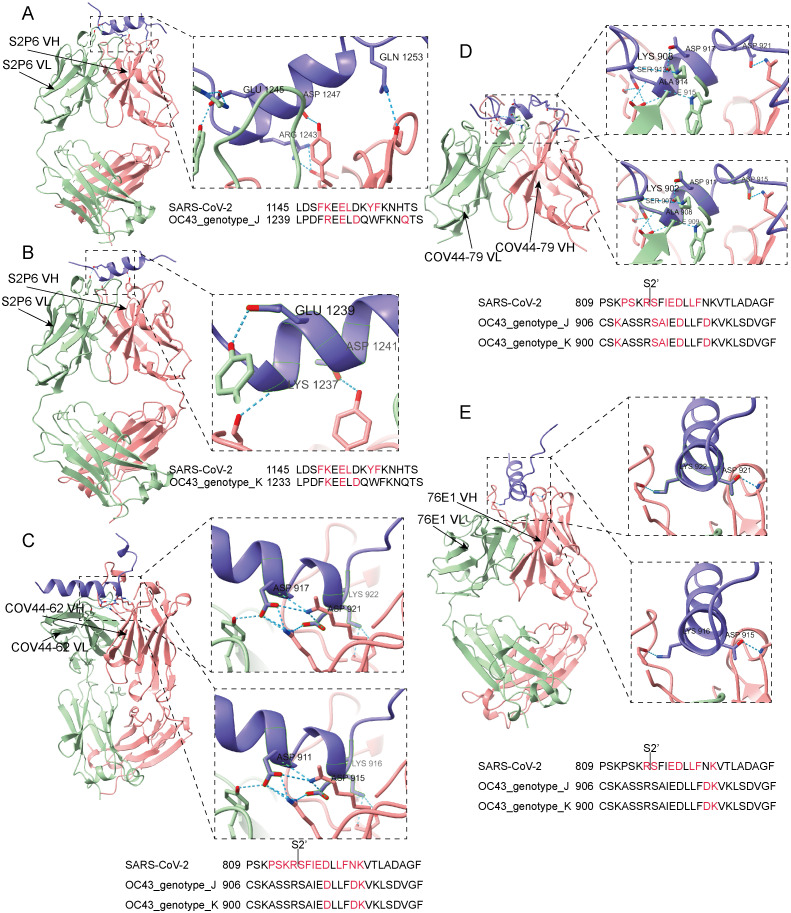
AlphaFold-predicted structures of HCoV-OC43 S protein antigen binding with broadly neutralizing antibodies against SARS-CoV-2. **A**. HCoV-OC43 genotype J Spike stem helix peptide binding to S2P6 antibody Fab fragment (PDB: 7NRJ). **B**. HCoV-OC43 genotype K Spike stem helix peptide binding to S2P6 antibody Fab fragment (PDB: 7NRJ). **C**. HCoV-OC43 Spike fusion peptide binding to COV44-62 antibody Fab fragment (PDB: 8D36). **D**. HCoV-OC43 Spike fusion peptide binding to COV44-79 antibody Fab fragment (PDB: 8DAO). **E**. HCoV-OC43 Spike fusion peptide binding to 76E1 antibody Fab fragment (PDB: 7X9E). Due to the identical spike fusion peptide sequences of genotypes J and K, both genotypes were analyzed together in panels **C**, **D**, and **E**. Each panel shows genotype J at the top and genotype K at the bottom. The heavy chain of the antibody Fab antigen-binding fragment is depicted in pink cartoons, and the light chain is shown in green cartoons. The viral binding peptide is represented in purple cartoons. Antigen-antibody hydrogen-bonding interaction residues are highlighted in stick atoms and annotated with text labels. Blue dashed lines indicate hydrogen bonds. The sites highlighted in red on the peptide sequences indicate antigen–antibody hydrogen-bonding interaction residues. SARS-CoV-2 interaction residues were annotated based on the related source literatures [21–23].

Additionally, focusing on four broadly neutralizing antibodies—S2P6, COV44–62, COV44–79, and 76E1—which were originally developed against SARS-CoV-2 but also exhibit cross-neutralization activity against HCoV-OC43, the antigen–antibody binding results showed that all four antibodies target relatively conserved regions in the S2 subunit of the S protein of HCoV-OC43. Specifically, S2P6 bound to the stem helix region, while COV44–62, COV44–79, and 76E1 bound to the fusion peptide region which includes the S2’ cleavage site (Fig 5). Detailed hydrogen bond interactions at the antigen–antibody interfaces are shown in Fig 5. Among these binding sites, sites 925 (921), 1247 (1243), and 1249 (1245) in genotype J, and sites 912 (902), 925 (915), 1247 (1237), 1249 (1239), and 1251 (1241) in genotype K were also identified as predicted conformational B-cell epitope residues (Fig 5 and S5 Table). Notably, site 1251 (1241) was also one of the previously identified positively selected sites (Fig 5 and S3 Table). Here, 925 (921) indicates that 921 represents the antigen–antibody interaction site, which also overlaps with the conformational epitope site predicted in this study, while 925 corresponds to the aligned site position in the original sequence used for selection pressure analysis. Furthermore, the stem helix and fusion peptide regions involved in antibody binding show a high degree of amino acid sequence similarity between SARS-CoV-2 and HCoV-OC43 (Fig 5). These findings, based on protein structural analyses of antigen–antibody interactions, provide structural evidence supporting potential cross-reactivity between SARS-CoV-2 and HCoV-OC43 within host cells.

## Discussion

In this study, we performed viral genotyping of HCoV-OC43 using phylogenetic analyses based on the WG, S, RdRp, and N gene sequences. Through the estimation and comparison of nucleotide substitution rates, we found an accelerated evolutionary trend in these genes of the currently dominant genotype K of HCoV-OC43, following the emergence of SARS-CoV-2. This finding suggests a potential shift in the evolutionary dynamics of HCoV-OC43, possibly influenced by changes in host immune landscapes or ecological pressures in the post-SARS-CoV-2 era.

A similar accelerating discovery was reported in a study on the genomic evolution analysis of human respiratory syncytial virus (HRSV) conducted by Maria Piñana et al. in 2024 [24]. They found that both HRSV-A and HRSV-B subtypes showed an increase in nucleotide substitution rates after the SARS-CoV-2 pandemic. Logically, the emergence of this accelerated trend of viral evolution is more plausible for the seasonal coronavirus HCoV-OC43, which is structurally similar to SARS-CoV-2 and belongs to the same genus, *Betacoronavirus*.

SARS-CoV-2 and HCoV-OC43 are similar in structural and virological characteristics, both are transmitted by respiratory droplet transmission or close contact, and both are capable of infecting humans. The competition between the two viruses for ecological niches may lead to competition for the same host or biological resources, which is exacerbated by the widespread implementation of social quarantine measures [25,26]. Moreover, SARS-CoV-2 shows greater transmission efficiency than HCoV-OC43. SARS-CoV-2 spread rapidly and globally upon its emergence. The infectiousness of SARS-CoV-2 was already estimated by the parameter basic reproduction number (R0), with an early R0 of about 2.2 and a mean R0 of 1.6-6.5; while the reported annual prevalence of endemic coronaviruses detected in hospital-based cohorts was only 6% on average, although most of these infections were detected as HCoV-OC43 [8,25,27–31]. Besides, due to frequent SARS-CoV-2 reinfections and widespread vaccinations, SARS-CoV-2 may indirectly contribute to the evolution of HCoV-OC43 mutations through host cross-reactivity. Several studies have found cross-reactivity between SARS-CoV-2 and seasonal coronaviruses in host cells [19,20]. Additionally, in this study, we confirmed the presence of SARS-CoV-2 and HCoV-OC43 cross-reactivity in host cells based on protein 3D structural evidence. Through predictive structural modeling, we successfully obtained the 3D structures of antigen-antibody complexes, identified the corresponding binding epitopes, and mapped specific interaction sites within these epitopes. Notably, a positively selected site (1251) was detected within the binding region. Therefore, our findings provide structural-level evidence supporting the existence of cross-reactivity between SARS-CoV-2 and HCoV-OC43 in host cells.

By performing selection pressure analysis and ancestral sequence reconstruction, we found that in genotype J, positively selected mutations are primarily concentrated in the CTD of the S1 subunit, and are mostly located on phylogenetic tree nodes corresponding to sequences collected before the SARS-CoV-2 pandemic. In contrast, genotype K exhibits positively selected mutations mainly in the NTD of the S1 subunit and the fusion peptide region of the S2 subunit, predominantly on nodes associated with post-pandemic sequences. Based on tMRCA, genotype J likely emerged earlier than genotype K. Notably, since 2024, all globally collected viral strains have belonged to genotype K, with no detection of genotype J. These suggest that genotype K currently holds the dominant status. Moreover, the accumulation of adaptive mutations at multiple positively selected sites in the S1 NTD, S2 fusion peptide region, and N protein, along with possible co-evolution among these regions (NTD-FP-N), may enhance the viral ability to recognize and bind to host cell sialic acid receptors, improve membrane fusion and viral replication/assembly efficiency, and ultimately facilitate more efficient host cell entry and transmission. In contrast, genotype J appears to have entered a relatively stable adaptive phase. After undergoing adaptive evolution, its major advantageous mutations may have already become fixed, leading to a more stable genetic composition within the population and a reduced evolutionary rate. Consequently, post-pandemic mutations in the S protein are likely to be neutral or under purifying selection, resulting in a slower accumulation of new beneficial mutations. We therefore propose that genotype J may have largely adapted to current host and environmental conditions and is presently in a relatively stable, low-variability evolutionary stage, though not in a state of complete stasis or absolute equilibrium.

The spike protein of coronaviruses is known for its high variability and frequent mutations [14,32,33], whereas the RdRp and N proteins are relatively conserved [14,32]. In genotype J, we observed that the S gene exhibited a decelerated evolutionary trend after the pandemic, while the RdRp and N genes evolved more rapidly. This contrasting pattern may reflect differences in selective pressures and functional constraints. The S protein, having undergone strong adaptive evolution during earlier host adaptation, may have reached a relatively optimized state for receptor binding and immune evasion, leading to a reduced rate of further advantageous mutations. By contrast, the more conserved RdRp and N genes may be experiencing compensatory or fine-tuning mutations to maintain replication efficiency and genomic stability in response to accumulated changes elsewhere in the viral genome.

Driven by the SARS-CoV-2 pandemic, global efforts to develop antiviral drugs targeting SARS-CoV-2 have accelerated significantly. These antivirals fall into two main categories: one targets viral proteins—primarily viral enzymes—to disrupt the viral life cycle, while the other targets host proteins involved in viral entry and replication, such as host cell receptors or proteases [34]. Several antiviral drugs that have been approved or are in late-stage clinical trials primarily target conserved viral domains, including Paxlovid (Nirmatrelvir/Ritonavir) [35], Molnupiravir (EIDD-2801/MK-4482) [36,37], and Remdesivir [38], which act on key viral enzymes such as the RNA-dependent RNA polymerase (RdRp/nsp12). These enzymes are highly conserved across coronaviruses and exhibit low tolerance to mutation, making them promising broad-spectrum antiviral targets. Notably, several studies have shown that Molnupiravir exhibits broad-spectrum activity against SARS-CoV-2, SARS-CoV, MERS-CoV, and bat-derived coronaviruses in human airway epithelial cell cultures [39] and humanized mouse models [40]. Moreover, some studies [41–43] indicate that coronaviruses tend to accumulate mutations more readily under drug pressure. Therefore, during the pandemic, the widespread use of broad-spectrum antiviral agents may have indirectly contributed to the accelerated evolution of structurally related viruses, such as HCoV-OC43.

The RdRp of coronaviruses possesses template-switching capability [44,45], which facilitates inter-template recombination during co-infection of a host by different HCoV-OC43 genotypes or closely related coronaviruses. This process can lead to genetic recombination and segment exchange, resulting in the continuous emergence and replacement of genotypes over time, thereby altering viral biological properties and antigenic structures, and enhancing adaptability and transmissibility [5,12]. In addition, during ongoing transmission, the S protein of HCoV-OC43 undergoes adaptive mutations under immune selection pressure [46]. As identified in this study, adaptive mutations at positively selected sites in the S1 NTD can increase the viral binding affinity for the host cell surface receptor 9-O-Ac-Sia. These mutations may facilitate immune evasion either by altering antigenic epitopes to escape recognition by neutralizing antibodies or by gradually accumulating point mutations that drive antigenic drift, enabling continual adaptation to host immune responses. Through genetic recombination, HCoV-OC43 generates novel genotypes; adaptive mutations enhance infectivity and immune evasion; and antigenic drift leads to continuous changes in epitope antigenicity. These mechanisms drive the viral evolutionary response to immune selection pressure and sustain its long-term circulation in the human population, forming the core dynamics underlying the persistent endemicity of HCoV-OC43.

This study has several limitations. Most of the data were derived from publicly available databases, and the currently limited number of HCoV-OC43 genomic sequences may not fully reflect the virus’s true genetic diversity and circulation patterns. Linking positively selected sites with epitopes requires highly precise analyses, which our current study may not fully achieve. Moreover, our B-cell epitope prediction and antigen-antibody binding analyses were based on static structural models of the spike protein, which cannot fully account for receptor-induced dynamic conformational changes. Due to current limitations in available resources and experimental data, dynamic conformational modeling of the HCoV-OC43 spike protein is not feasible for us. Therefore, our work provides a preliminary exploration of potential associations between positively selected sites and predicted epitopes, highlighting possible patterns rather than definitive correlations. Future studies could refine these analyses by mapping individual positively selected sites onto experimentally validated epitopes, integrating structural and antigenicity data, or applying statistical models to quantify site-by-epitope correlations more rigorously. Furthermore, large-scale sequencing combined with experimental validation—such as neutralization assays to assess cross-reactive antibody responses, protein functional assays to evaluate the effects of specific mutations, and comparative studies of viral adaptability to different hosts—will be essential to clarify the biological relevance of the predicted cross-reactivity and adaptive evolutionary patterns.

In conclusion, our findings suggest that the SARS-CoV-2 pandemic may have promoted the evolution of HCoV-OC43. Under selection pressure, adaptive mutations at key amino acid sites in the spike and nucleocapsid proteins of HCoV-OC43 may enhance the virus’s ability to recognize and bind to host sialic acid receptors, facilitate membrane fusion and viral assembly, and promote efficient host cell entry. These evolutionary adaptations likely contribute to viral persistence and ecological competitiveness. Additionally, immune pressure resulting from cross-reactivity between SARS-CoV-2 and HCoV-OC43 in host cells may serve as a driving force for the accelerated evolution of HCoV-OC43.

## Methods

### Data download and preprocessing

The data for this study were sourced from two parts. One part consisted of complete genome sequences of HCoV-OC43 obtained by sequencing. Based on the 2024 comprehensive surveillance of acute respiratory infections in Jing’an District, Shanghai, we used the Real-Time PCR Diagnostic Kit Rapid Detection of multiple pathogens of national acute respiratory infectious diseases (SMS-D404AAYF-C-10T-01K) (Beijing Zhuocheng Huisheng Biotechnology Co., Ltd., China) and the real-time quantitative PCR instrument LightCycler 480 II (F. Hoffmann-La Roche Ltd., Basel, Switzerland) for multi-pathogen detection. This kit contains specific primers and fluorescent probes for detecting the corresponding pathogen genes. By collecting fluorescence signals during the PCR amplification process, we monitored whether S-shaped amplification and Ct values ≤38 were present in different detection channels, which allowed for the qualitative detection of multiple respiratory pathogens, including four subtypes of HCoVs. A total of 85 HCoV-positive samples were detected, including 6 cases of HCoV-229E, 43 of HCoV-NL63, 28 of HCoV-OC43, and 11 of HCoV-HKU1. Due to co-infection between different subtypes, the total number of positive cases did not equal the sum of individual subtypes. Finally, based on sample quality and retesting of Ct values, we successfully sequenced 14 whole genomes of HCoV-OC43 using the Illumina Viral Surveillance Panel v2 (VSP2) reagent kit (hybrid capture enrichment method) and the Illumina MiSeq platform. Reference sequence-guided assembly was performed with MEGAHIT v1.1.3 [47] and Ragtag v2.1.0 [48]. These sequences were aligned and trimmed according to the reference sequence NC_006213.1 to extract the S, RdRp, and N gene segments, which were subsequently incorporated into the collected sequence datasets. All 14 sequences obtained have been submitted to GenBank with the accession numbers: PV798427-PV798440.

Additionally, the other part comprised the WG, S, RdRp, and N gene sequences of HCoV-OC43 downloaded from the National Center for Biotechnology Information (NCBI) database (https://www.ncbi.nlm.nih.gov) on January 15, 2025. Among them, we excluded sequences for which no collection date could be reliably obtained either through the NCBI database or publications, sequences with too many ambiguous bases (>1% of the total sequence length), sequences with consecutively long Ns (more than 10 consecutive Ns), sequences with obvious anomalies, and sequences shorter than 90% of the respective gene or whole genome length. However, considering that the number of sequences collected post-pandemic was less than that of pre-pandemic, we appropriately relaxed the restriction on the occurrence of consecutive unknown base N in sequences collected post-pandemic.

After data preprocessing, we finally obtained four sequence datasets of the WG, S, RdRp, and N of the HCoV-OC43. The WG dataset included 440 sequences in total, with 368 and 72 sequences collected pre-pandemic and post-pandemic; the S dataset included 621 sequences in total, with 545 and 76 sequences collected pre-pandemic and post-pandemic; the RdRp dataset included 655 sequences in total, with 569 and 86 sequences collected pre-pandemic and post-pandemic; the N dataset included 725 sequences in total, with 637 and 88 sequences collected pre-pandemic and post-pandemic.

### Phylogenetic analysis and HCoV-OC43 genotyping

Each dataset described above was subjected to multiple sequence alignment using MAFFT v7.520 [49] and manual adjustment using MEGA v7.0 [50]. Subsequently, the maximum likelihood (ML) trees were constructed with the bootstrap set to 5000 in IQ-TREE v2.2.0 (integrated ModelFinder) [51], and several sequences of bovine coronavirus (BCoV) were used as the outgroup sequences to clarify the phylogenetic relationships. HCoV-OC43 genotyping was performed based on the reference typing sequences and phylogenetic relationships, using the naming convention proposed by Lau et al [12]. All phylogenetic trees were embellished using the online tool iTOL [52]. The reference typing sequences were summarized and organized based on the available articles [5,12,53–59] about HCoV-OC43 genotyping of the WG, S, RdRp, and N genes. Specific reference typing information for these sequences is provided in S6 Table.

### Bayesian evolutionary analysis

To explore the changes in nucleotide substitution rates of HCoV-OC43 before and after the SARS-CoV-2 pandemic, as well as the time to the most recent common ancestor (tMRCA), analyses were conducted in BEAST software on the predominant genotypes. The nucleotide substitution rate reflects the evolutionary rate, while tMRCA suggests the earliest time of origin. Based on the genotyping results of the WG, S, RdRp, and N of HCoV-OC43, we generated several datasets of all, the pre-pandemic, and the post-pandemic sequences for the predominant genotypes. For each dataset, we utilized MAFFT v7.520 to conduct multiple sequence alignment, followed by manual adjustment using MEGA v7.0. BEAST v1.10.4 [60] was used to estimate the rate of nucleotide substitutions per site per year and tMRCA using the Bayesian Markov Chain Monte Carlo (MCMC) method, and to generate the maximum clade credibility (MCC) tree. The best-fit site substitution model was identified based on the Bayesian Information Criterion (BIC) using IQ-TREE v2.2.0 (integrated ModelFinder, the bootstrap set to 5000). TreeTime v0.11.3 [61] was used to evaluate the temporal signal of the datasets to remove sequences with aberrant temporal signal. Referring to parameter settings in the existing research [12], all BEAST analyses were conducted independently using a relaxed molecular clock model with an uncorrelated exponential distribution and a constant coalescent prior. The collection dates of sequences were used for molecular clock calibration. The number of total generations varied with the datasets used in each analysis, and the output samples were all no less than 10,000. Subsequently, the produced log file was imported into Tracer v1.10.4 [62] to assess and view the convergence of the chains. All parameters were estimated with an Effective Sample Size (ESS) over 200, indicating sufficient convergence. Besides, the MCC tree was inferred with a burn-in value set to 10% using TreeAnnotator v1.10.4 [60] and visualized in Figtree v1.4.4 [60]. In addition, to exclude the potential impact of recombination on the comparison of nucleotide substitution rates, we constructed the representative sequence dataset for different genotypes of HCoV-OC43 and performed recombination analyses. The detailed recombination methodology is provided in the S1 File.

### Selection pressure analysis and historical evolution analysis of positively selected sites

Selection pressure was evaluated for the predominant genotypes of S, RdRp, and N genes of HCoV-OC43 using the Fixed Effects Likelihood (FEL) [63], Mixed Effects Model of Evolution (MEME) [64], Single-Likelihood Ancestor Counting (SLAC) [63], and Fast Unconstrained Bayesian Approximation (FUBAR) [65] methods in HyPhy v2.5.62 [66]. Positively selected sites were identified based on statistical significance (p-value < 0.1 in FEL, MEME, and SLAC or posterior probability < 0.9 in FUBAR) by at least one method.

After selection pressure analysis, the nucleotide sequences of the S gene with positive selection sites were translated to amino acid sequences using MEGA v7.0. Then, the amino acid sequences and the related MCC tree generated after the BEAST analysis were imported together into TreeTime v0.11.3 for ancestral sequence reconstruction. Finally, we marked the mutations at the positively selected sites on the tree nodes of the previously constructed MCC tree, observing the historical evolution pattern of positively selected mutation sites.

### Polymorphism analysis of amino acid variation sites

The S gene sequences of the predominant genotypes of HCoV-OC43 before and after the pandemic were aligned, adjusted, and translated into protein amino acid sequences, followed by a second alignment and adjustment. The majority consensus amino acid sequences were generated using Lasergene v11.1 MegAlign software from DNASTAR, Inc. All alignments and adjustments were performed with MAFFT v7.520 and MEGA v7.0. By comparing the majority consensus amino acid sequences before and after the pandemic, amino acid variation sites were identified, and the amino acid usage frequencies at these sites were calculated. Chi-square tests were performed using IBM SPSS Statistics for Windows v25.0 to evaluate whether the differences in the usage frequencies of major amino acids at variable sites before and after the pandemic were statistically significant.

### Prediction of potential linear and conformational B-cell epitopes

Proceed with the same operation as before to obtain the majority consensus amino acid sequence of the full-length S protein of the predominant genotypes. Protein secondary structure was predicted using the online server SOPMA (https://npsa.lyon.inserm.fr/cgi-bin/npsa_automat.pl?page=/NPSA/npsa_sopma.html) with the number of conformational states set to 4 (Helix, Sheet, Turn, Coil) and other parameters defaulted. Protein parameters, including hydrophobicity, flexibility, antigenic index, and surface probability, were predicted using Lasergene v11.1 Protean software from DNASTAR, Inc. Linear B-cell epitopes were predicted using the online server BepiPred-3.0 (https://services.healthtech.dtu.dk/services/BepiPred-3.0/) with higher confidence (top 20%). Finally, potential linear B-cell epitopes were identified as those predicted epitopes satisfying the following two conditions: not within alpha-helix or beta-bridge regions of the protein secondary structure; in the regions with flexibility > 0, antigenic index > 0, surface probability > 0, and hydrophobicity < 0.

Based on the majority consensus amino acid sequences, the three-dimensional (3D) structure of the spike protein was predicted using the AlphaFold server (https://alphafoldserver.com/). Conformational B-cell epitopes were subsequently predicted from the 3D structure using the DiscoTope-3.0 server (https://services.healthtech.dtu.dk/services/DiscoTope-3.0/) with default parameters.

### Antigen–antibody binding analysis

The cryo-EM-resolved 3D structure of the HCoV-OC43 spike protein in complex with the host cell 9-O-Ac-Sia sialic acid receptor (PDB: 6NZK) was obtained from the Protein Data Bank (PDB, https://www.rcsb.org/) and analyzed for receptor-binding regions based on the 3D structure and the source literature [67]. For antibodies against SARS-CoV-2 with broadly neutralizing activity but lacking resolved antigen–antibody complex structures with HCoV-OC43, antigen–antibody binding analyses were performed using the AlphaFold server based on amino acid sequences. Representative antibodies analyzed in this study included S2P6 [21], COV44–62 [22], COV44–79 [22], and 76E1 [23], referring to the related source literature [21–23] and the antigen–antibody complexes available in the PDB (PDB: 7RNJ, 8D36, 8DAO, and 7X9E). Protein structure visualization and annotation were conducted using ChimeraX v1.8 [68].

## Supporting information

S1 Fig Maximum-likelihood phylograms of the predominant genotypes J and K. A-D. Maximum-likelihood (ML) trees based on the WG, S, RdRp, and N genes of HCoV-OC43 genotype K (expressed at the N gene level as genotype I) (A. WG ML tree; B. S ML tree; C. RdRp ML tree; D. N ML tree). E-H. ML trees based on the WG, S, RdRp, and N genes of HCoV-OC43 genotype J (E. WG ML tree; F. S ML tree; G. RdRp ML tree; H. N ML tree). Branch lengths are proportional to the number of substitutions per site, allowing visualization of relative genetic distances among sequences.
(PDF)

S2 Fig Potential linear B-cell epitopes of the predominant genotypes J and K on the S protein of HCoV-OC43. A. potential linear B-cell epitopes of genotype J on the S protein. B. potential linear B-cell epitopes of genotype K on the S protein. The letter h in blue lowercase indicates the alpha helix. The letter e in red lowercase indicates the extended strand. The letter t in green lowercase indicates the beta turn. The letter c in yellow lowercase indicates the random coil. The orange underlining indicates the peptide predicted by BepiPred-3.0. The green box indicates the peptide predicted by SOPMA. The red box indicates the peptide predicted by Protean.
(TIF)

S3 Fig Structural analysis of HCoV-OC43 binding to the 9-O-Ac-Sia receptor based on the PDB database. A. the 9-O-Ac-Sia receptor binding region on the S protein of HCoV-OC43 (PDB: 6NZK). B-C. panels B and C show enlarged views of selected areas in panel A. Hydrogen-bonding interaction residues are highlighted in orange. Blue dashed lines indicate hydrogen bonds. The structural interpretations referenced the research of Tortorici, M. A. et al [67].
(TIF)

S1 Table Summary of the same virus strains of HCoV-OC43 based on WG, S, RdRp, and N genotyping. (XLSX)

S2 Table Nucleotide substitution rates and tMRCA of the HCoV-OC43 predominant genotypes. (XLSX)

S3 Table Sites under positive selection identified by selection pressure analysis of HCoV-OC43. (XLSX)

S4 Table Summary of potential linear B-cell epitopes in the spike protein of HCoV-OC43. (XLSX)

S5 Table Summary of potential conformational B-cell epitopes in the spike protein of HCoV-OC43. (XLSX)

S6 Table The reference genotypes of HCoV-OC43 in this study from references. (XLSX)

S1 File Recombination analysis. (DOCX)
