# Supplementary figures and images for: Pandemic-driven immune imprinting accelerates evolution of human coronavirus OC43
Source: PLoS Negl Trop Dis. 2026 Mar 17;20(3):e0014109. doi: 10.1371/journal.pntd.0014109 (PMC12994797; doi:10.1371/journal.pntd.0014109)

A WG\_genotype\_K

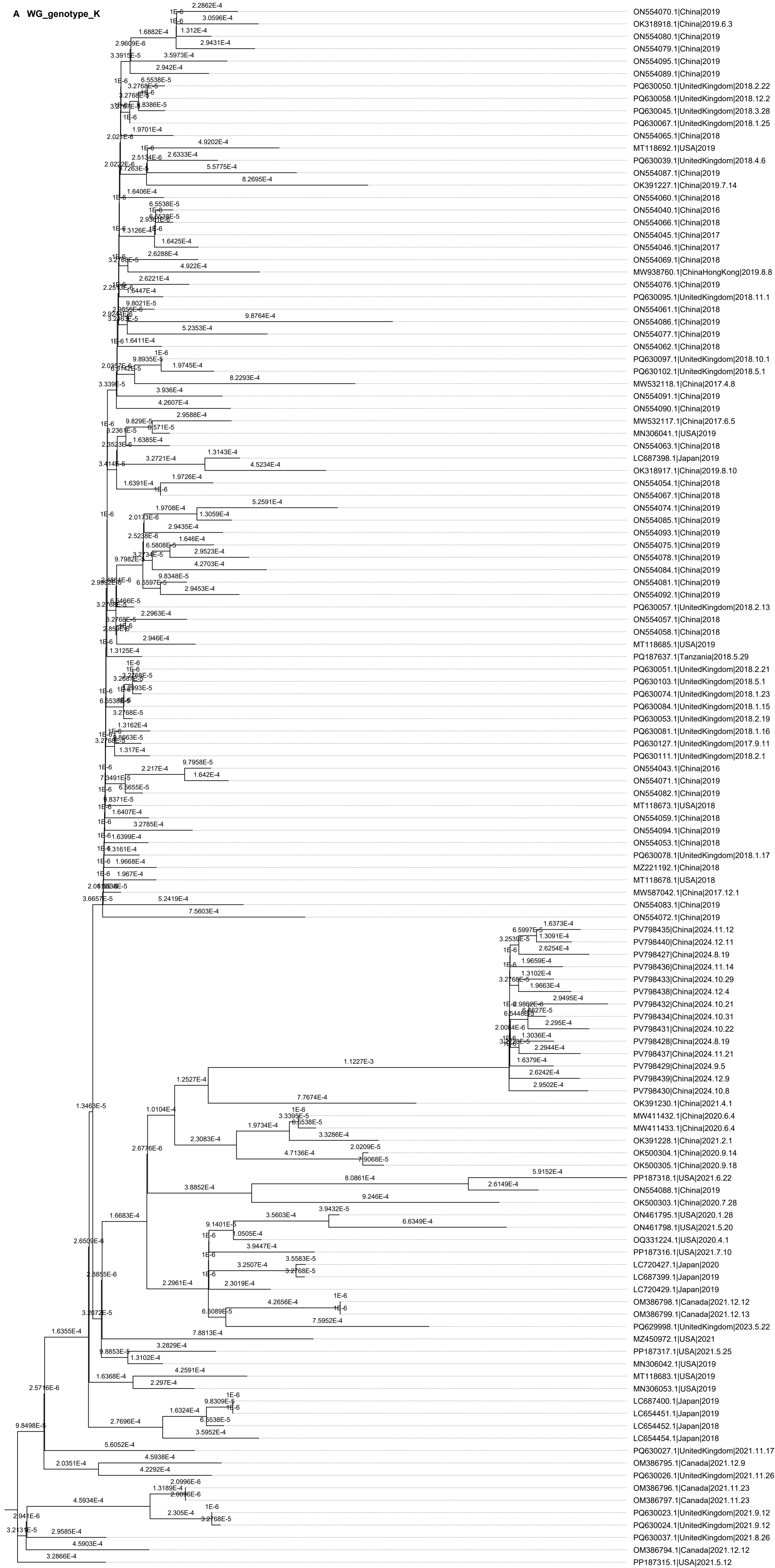

3.0E-4

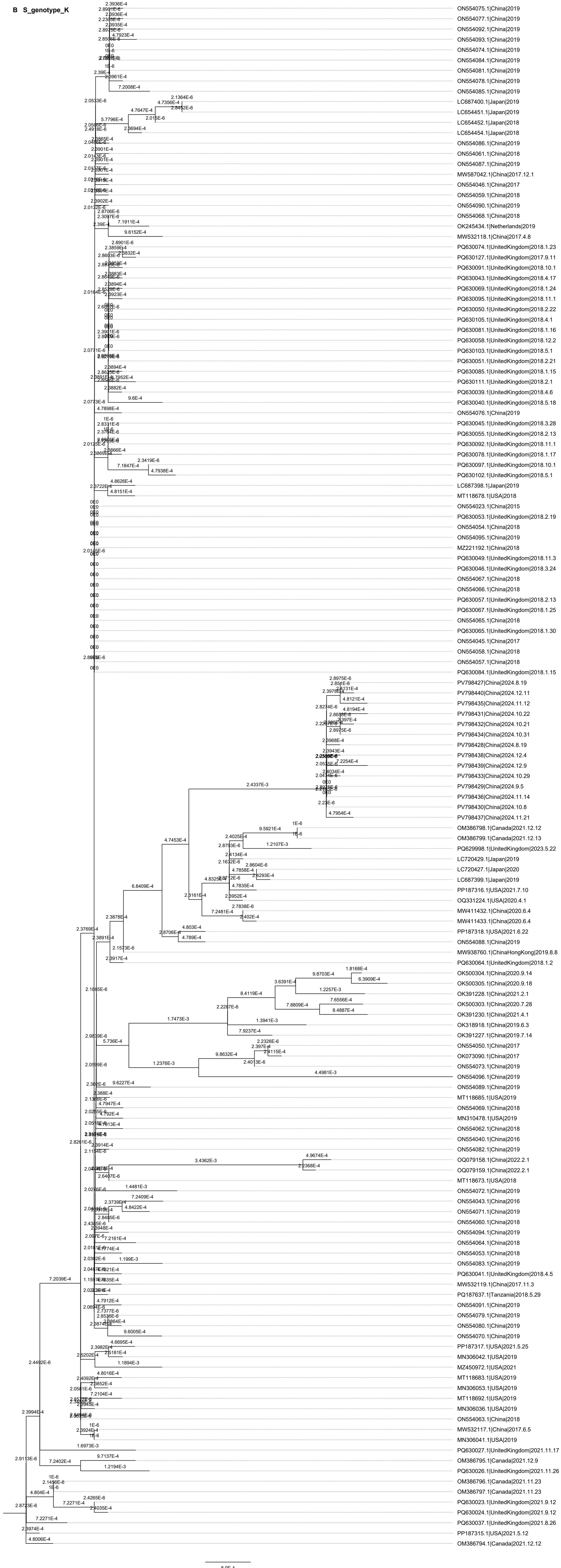

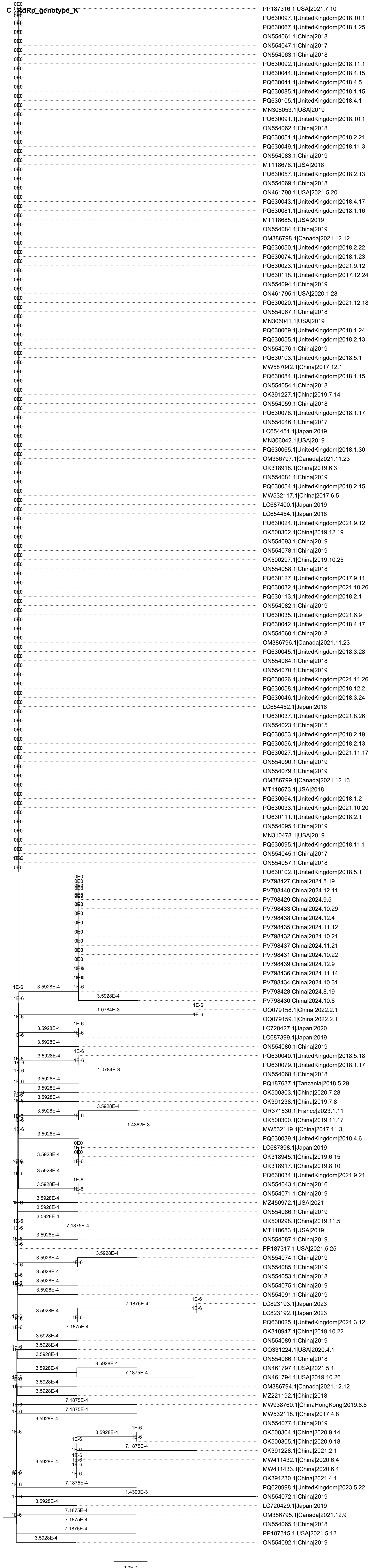

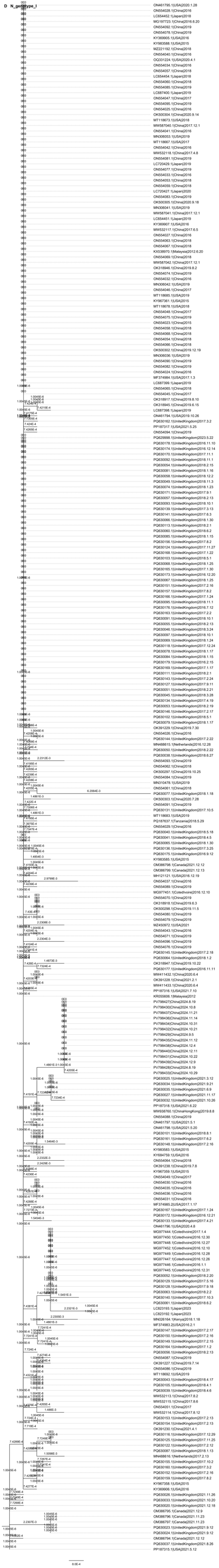

E WG\_genotype\_J

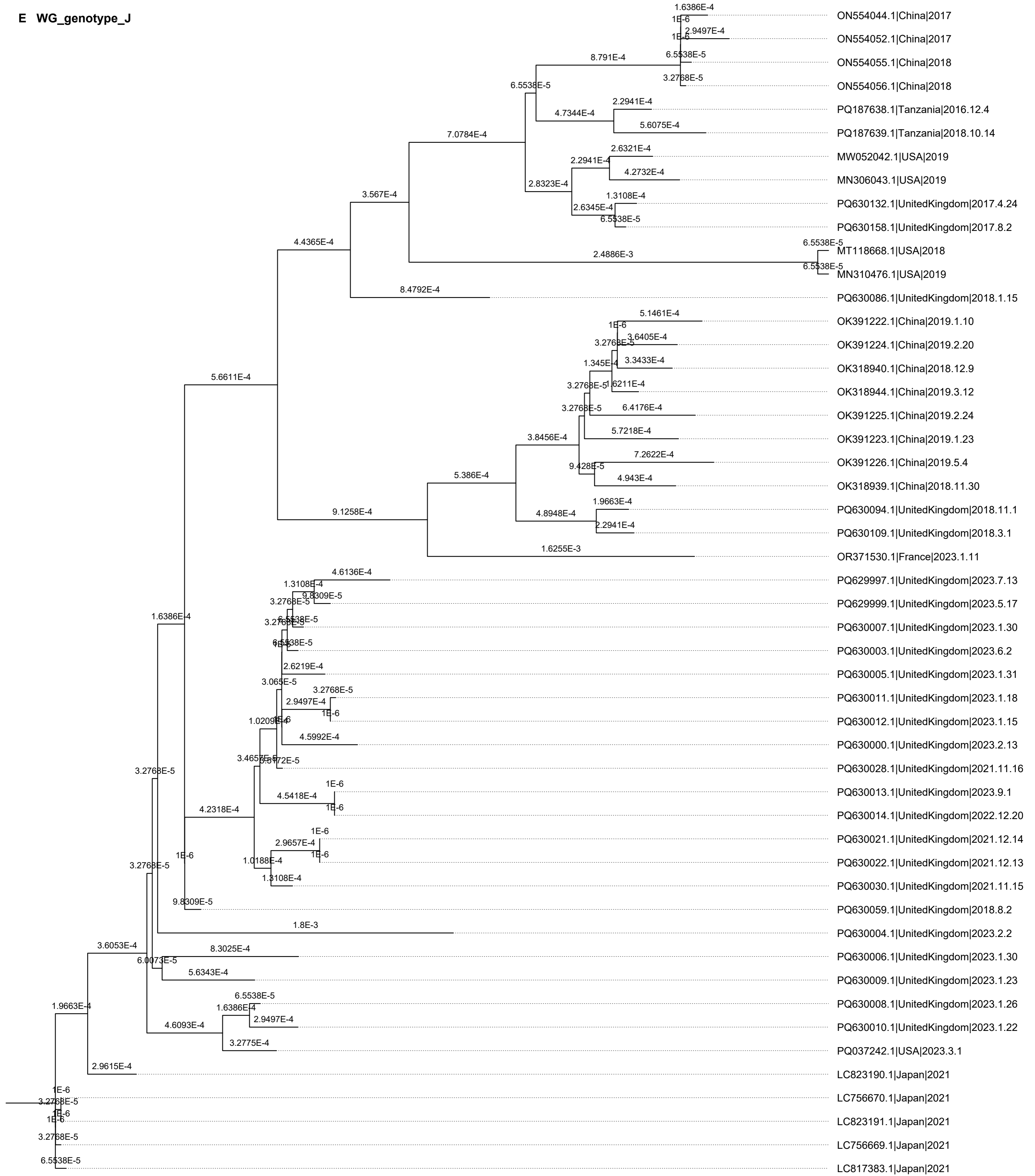

F S\_genotype\_J

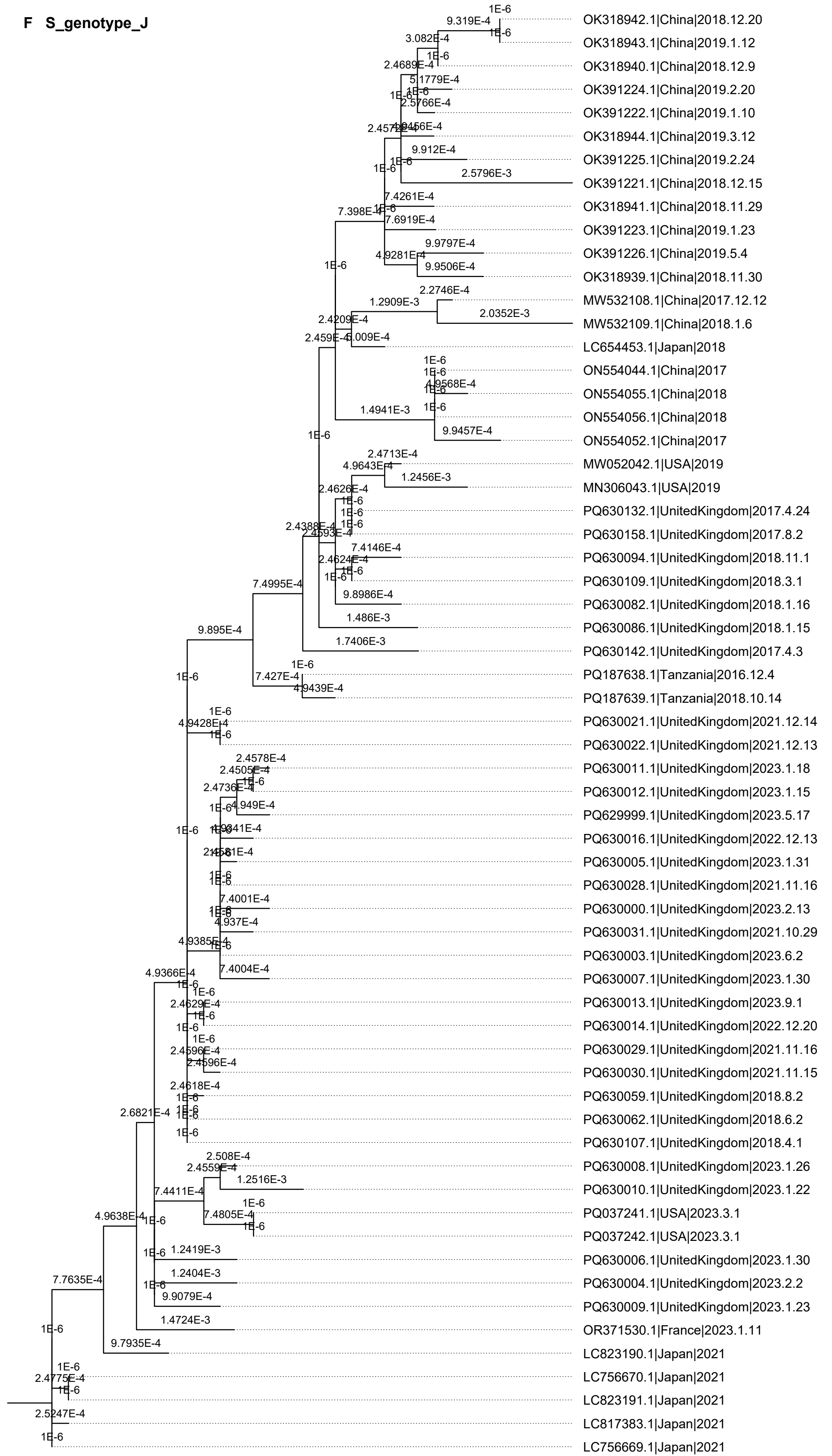

8.0E-4

G RdRp\_genotype\_J

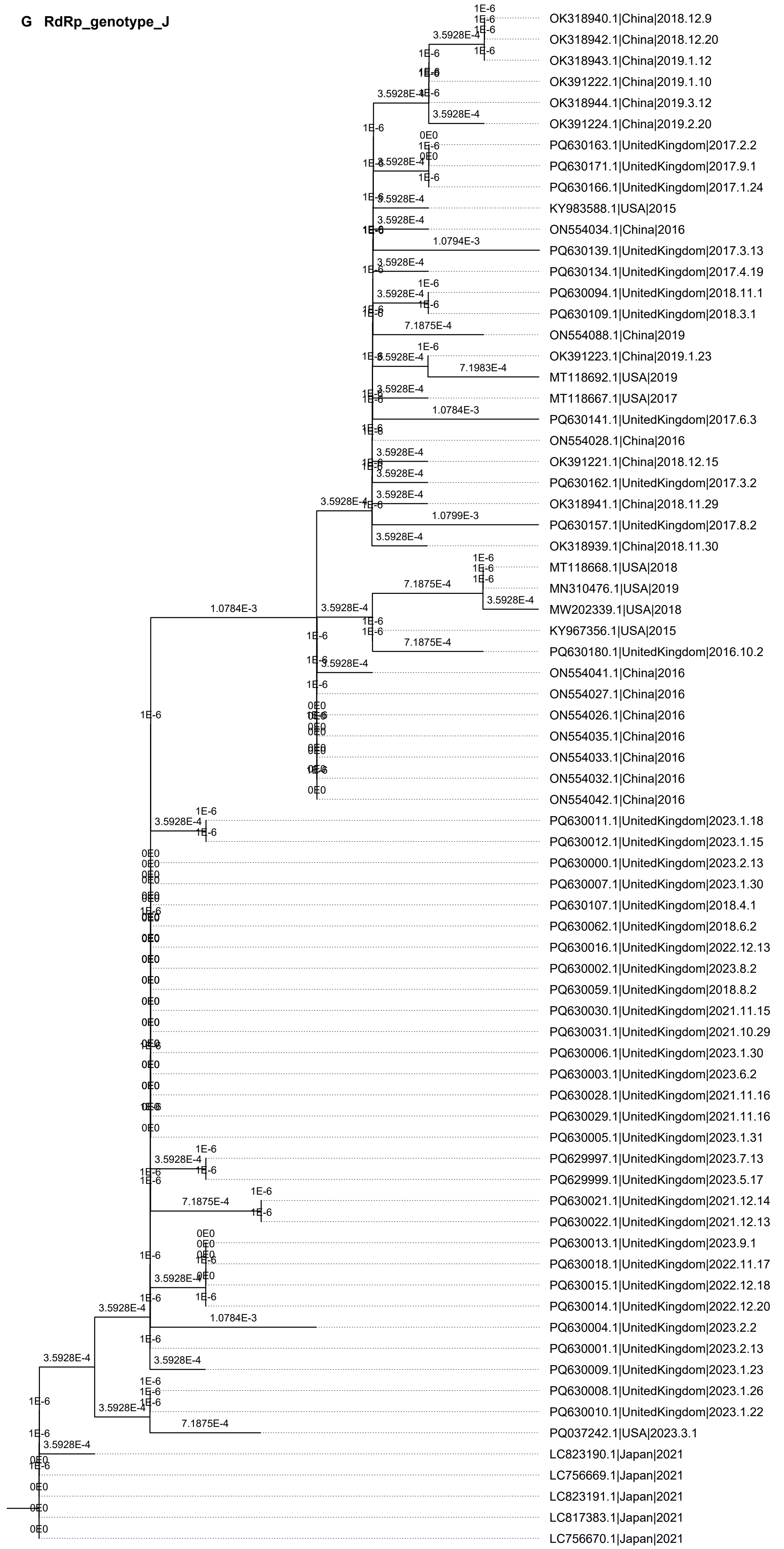

4.0E-4

H N\_genotype\_J

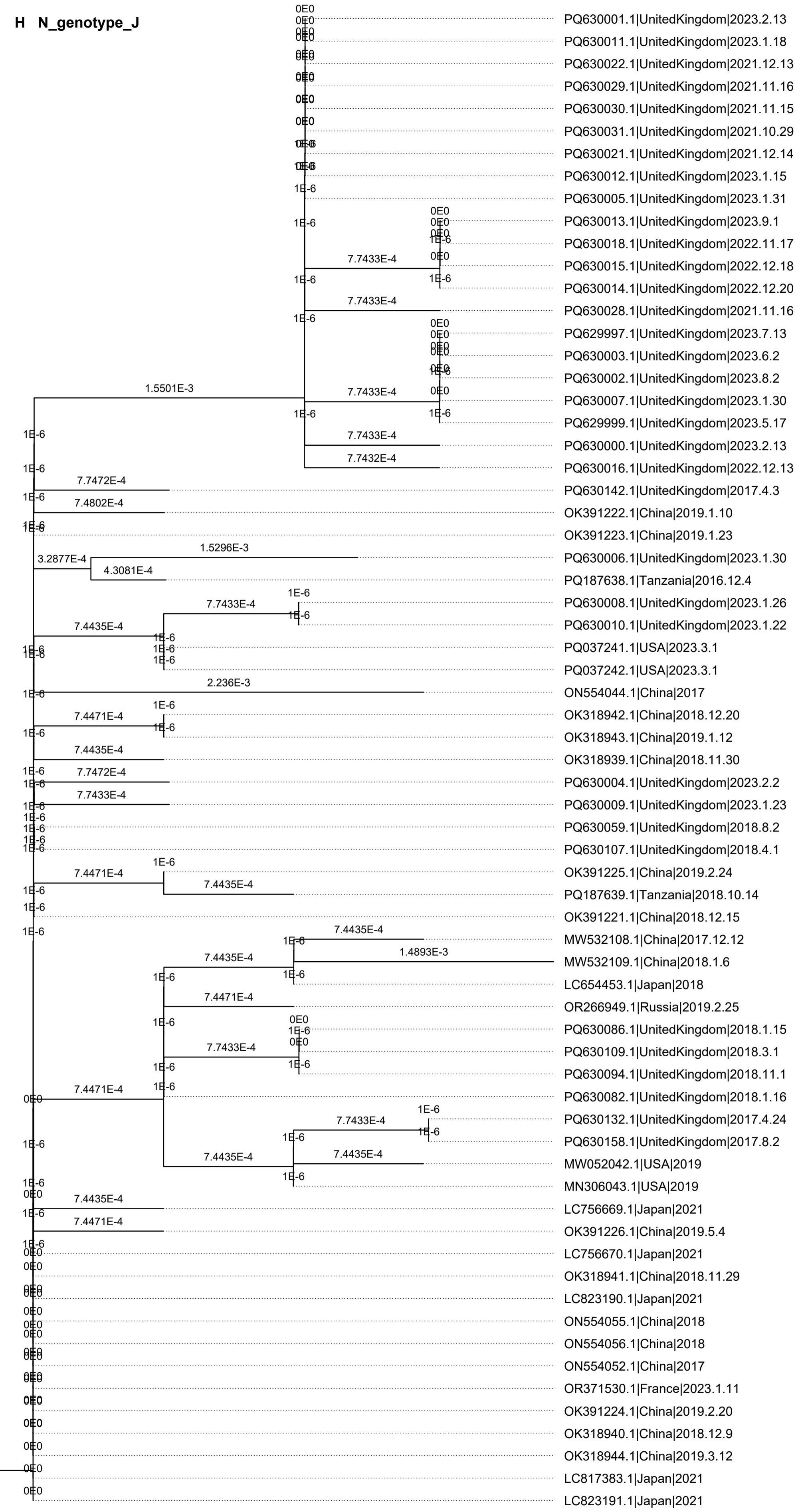

3.0E-4

Supplement: S1 Fig — A-D. Maximum-likelihood (ML) trees based on the WG, S, RdRp, and N genes of HCoV-OC43 genotype K (expressed at the N gene level as genotype I) (A. WG ML tree; B. S ML tree; C. RdRp ML tree; D. N ML tree). E-H. ML trees based on the WG, S, RdRp, and N genes of HCoV-OC43 genotype J (E. WG ML tree; F. S ML tree; G. RdRp ML tree; H. N ML tree). Branch lengths are proportional to the number of substitutions per site, allowing visualization of relative genetic distances among sequences. (PDF) [file pntd.0014109.s001.pdf]

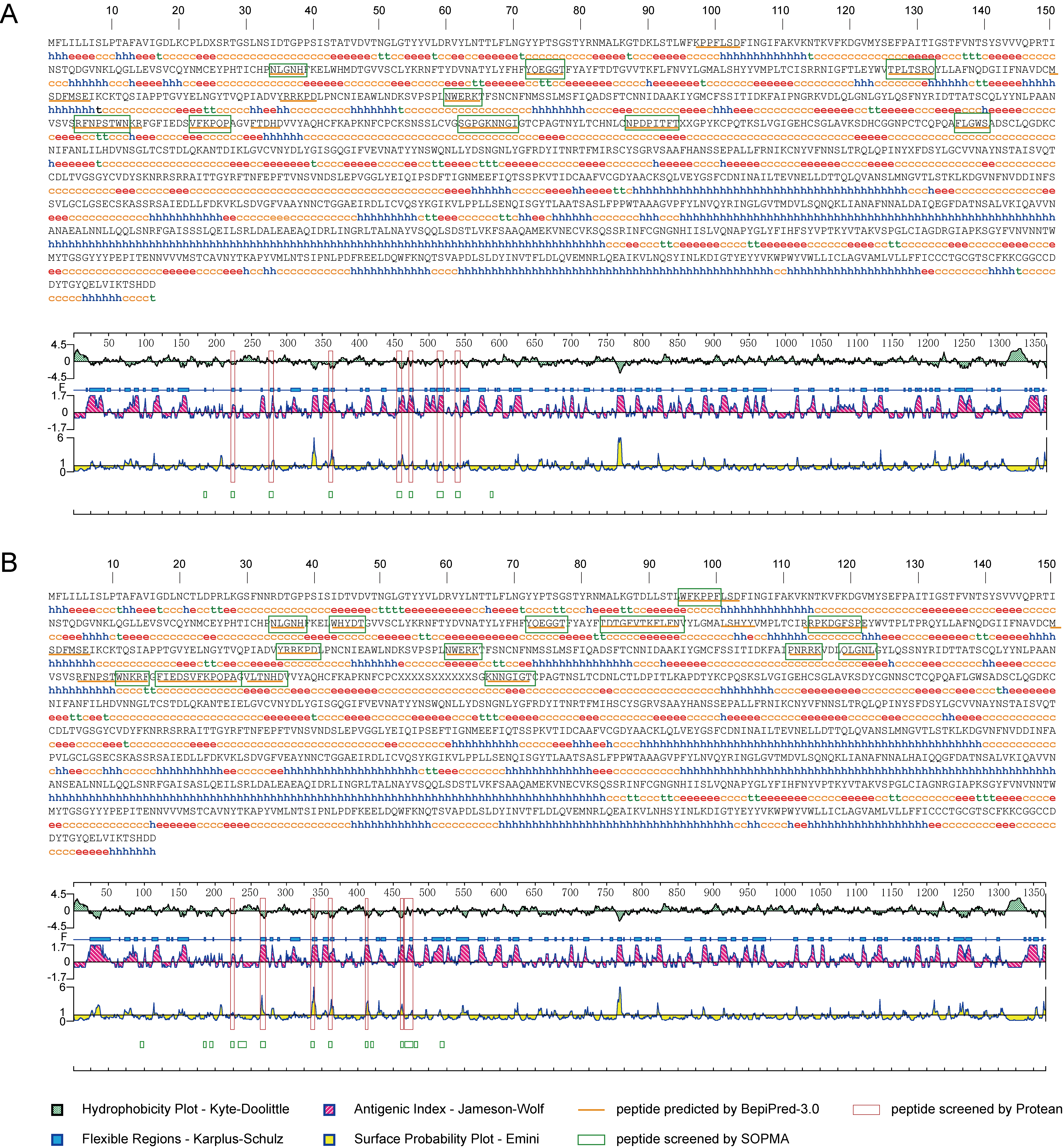

Supplement: S2 Fig — A. potential linear B-cell epitopes of genotype J on the S protein. B. potential linear B-cell epitopes of genotype K on the S protein. The letter h in blue lowercase indicates the alpha helix. The letter e in red lowercase indicates the extended strand. The letter t in green lowercase indicates the beta turn. The letter c in yellow lowercase indicates the random coil. The orange underlining indicates the peptide predicted by BepiPred-3.0. The green box indicates the peptide predicted by SOPMA. The red box indicates the peptide predicted by Protean. (TIF) [file pntd.0014109.s002.tif]

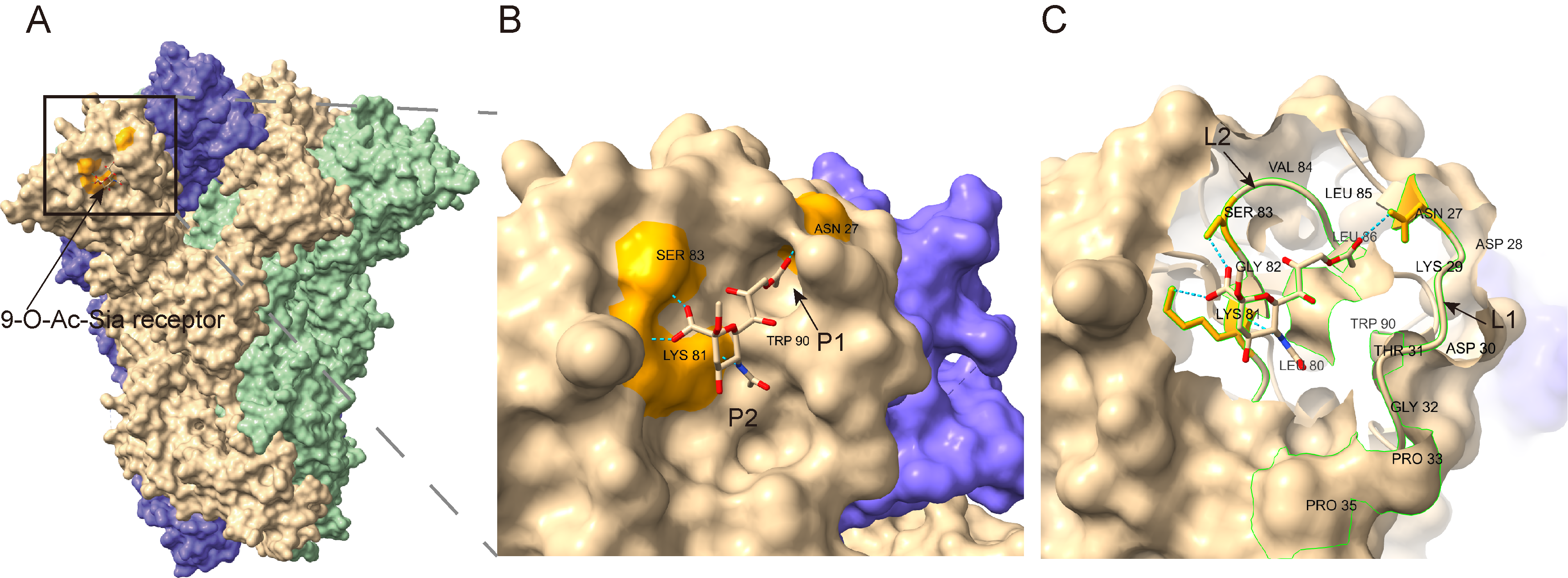

Supplement: S3 Fig — A. the 9-O-Ac-Sia receptor binding region on the S protein of HCoV-OC43 (PDB: 6NZK). B-C. panels B and C show enlarged views of selected areas in panel A. Hydrogen-bonding interaction residues are highlighted in orange. Blue dashed lines indicate hydrogen bonds. The structural interpretations referenced the research of Tortorici, M. A. et al [67]. (TIF) [file pntd.0014109.s003.tif]
