# Supplementary material for: Pandemic-driven immune imprinting accelerates evolution of human coronavirus OC43
Source: PLoS Negl Trop Dis. 2026 Mar 17;20(3):e0014109. doi: 10.1371/journal.pntd.0014109 (PMC12994797; doi:10.1371/journal.pntd.0014109)
Supplement: S1 File — (DOCX) [file pntd.0014109.s010.docx]

**S1 File. Recombination analysis**

**Recombination analysis methods:**

To exclude the potential impact of recombination on the comparison of nucleotide substitution rates in BEAST analysis, we constructed the representative sequence dataset for different genotypes of HCoV-OC43 and performed recombination analysis. Considering that using all sequences for recombination detection may introduce false positives and obscure parental lineage identification, we constructed a representative sequence dataset covering the 11 genotypes based on the whole-genome dataset and the whole-genome ML phylogenetic tree.

The selection criteria were as follows:(1) Genotypes J and K: All sequences of genotypes J or K were first de-redundified using CD-HIT v4.8.1 (similarity threshold: c = 0.995). Representative sequences were then supplemented according to phylogenetic relationships inferred from the ML tree. Specifically, we retained sequences phylogenetically adjacent to other genotypes, included at least one early sampling sequence and one late sampling sequence, preserved at least two sequences from both pre- and post-pandemic periods, and retained at least one sequence from each sub-branch with bootstraps ≥70. Approximately 10-25 representative sequences were selected per genotype. (2) Genotypes A-I: We retained sequences phylogenetically close to genotypes J or K, included at least one early sampling sequence and one late sampling sequence, and preserved at least one sequence from each sub-branch with bootstraps ≥70. Approximately 2-5 representative sequences were selected per genotype.

The representative sequence dataset of the 11 HCoV-OC43 genotypes was imported into RDP v4, and recombination screening was conducted using seven detection methods (RDP, GENECONV, BootScan, MaxChi, Chimaera, SiScan, and 3Seq). A potential recombination event for genotypes J or K was defined when a J/K sequence was identified as the recombinant, not as a major or minor parent, and was supported by at least three methods. Subsequently, the putative recombinant sequences and their parent sequences were analyzed in SimPlot v3.5.1 for validation, assessing whether clear and stable parental fragment exchanges were present.

**RDP and Simplot results:**

From the RDP results, only two sequences of genotype J (J_MN310476.1|USA|2019 and J_PQ630132.1|UnitedKingdom|2017) and one sequence of genotype K (K_LC654451.1|Japan|2019) were identified as potential recombinants, and all three were sampled in the pre-pandemic period. However, SimPlot validation did not reveal any clear or stable parental fragment exchanges.

**Table RDP. Recombination events detected by multiple methods for representative HCoV-OC43 genotypes**

| Event no. | Found in* | Recombinant | Major parent | Minor parent | Detection Methods | Beginning breakpoint | Ending breakpoint | Red highlight reminder in RDP |
| --- | --- | --- | --- | --- | --- | --- | --- | --- |
| 3 | 1 | J_MN310476.1 (2019) | F_PQ187629.1 | B_KF923888.1 | 6 methods | 90 | 23242 | No |
| 12 | 8 | J_PQ630132.1 (2017) | C_KF530063.1 | F_PQ187629.1 | 5 methods | Undetermined (29985) | 19643 | Yes, possible misidentification of recombinant |
| 14 | 14 | K_LC654451.1 (2019) | G_PQ630129.1 | E_KF530074.1 | 4 methods | Undetermined (24859) | 24989 | Yes, possible misidentification of recombinant |

* "Found in" indicates the number of sequences in the dataset in which this recombination event was detected.


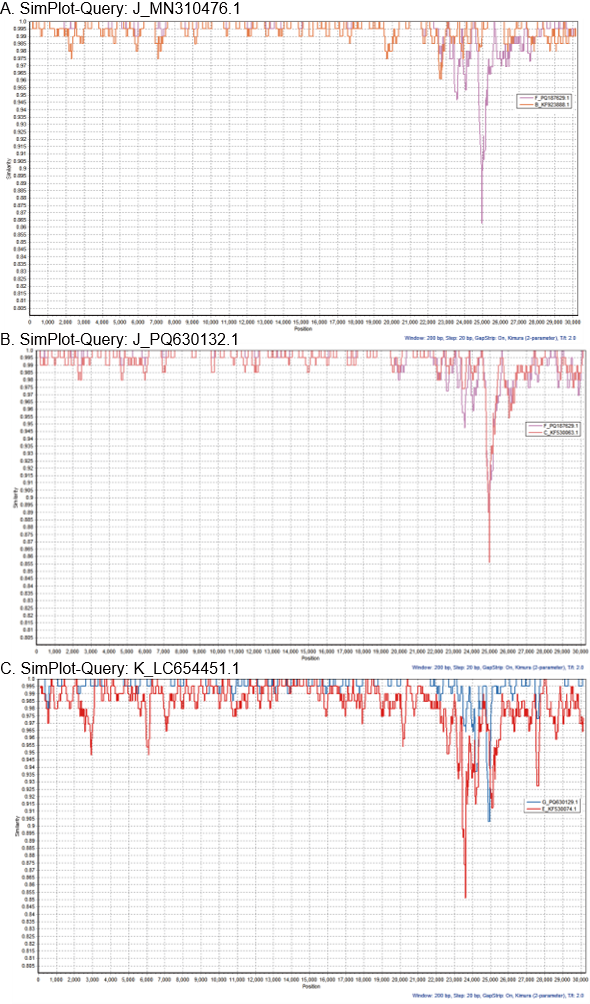


**Fig Simplot. Verification of recombination events in representative HCoV-OC43 sequences by SimPlot**. **A-C**, SimPlot results using three putative recombinant HCoV-OC43 sequences (J_MN310476.1, J_PQ630132.1, and K_LC654451.1) as query sequences, respectively.
